# Reviewer acknowledgement 2014

**DOI:** 10.1186/s40413-015-0056-1

**Published:** 2015-02-14

**Authors:** Alessandro Fiocchi, Erika Jensen-Jarolim

**Affiliations:** Hospital Bambino Gesù in Rome, Rome, Italy; Medical University Vienna and Messerli Research Institute, Vienna, Austria

## Abstract

The editors of *World Allergy Organization Journal* would like to thank all of our reviewers who have contributed to the journal in Volume 7 (2014).

**Mona Al-Ahmad**

Kuwait

**Suleiman Al-Hammadi**

United Arab Emirates

**Maria Cristina Artesani**

Italy

**Amal Assa'ad**

United States of America

**Emel Aygören-Pürsün**

Germany

**Claus Bachert**

Belgium

**Leonard Bielory**

United States of America

**W.M. Blom**

Netherlands

**Matteo Bonini**

Italy

**Homer Boushey**

United States of America

**Malcolm Brinn**

Australia

**Wesley Burks**

United States of America

**Carlo Caffarelli**

Italy

**Moises A. Calderon**

United Kingdom

**Giorgio Walter Canonica**

Italy

**Yoon-Seok Chang**

South Korea

**Marco Cicardi**

Italy

**Philip Cooper**

Ecuador

**Emily Cope**

United States of America

**Alexessander Couto Alves**

United Kingdom

**Linda Cox**

United States of America

**Julian Crane**

New Zealand

**Ceyhun Dalkan**

Cyprus

**Martin Depner**

Germany

**Martin Desrosiers**

Canada

**Ratko Djukanovic**

United Kingdom

**Sten Dreborg**

Sweden

**Motohiro Ebisawa**

Japan

**Alison Elliott**

Uganda

**Ira Finegold**

United States of America

**Puccio Franca**

Venezuela

**Maia Gotua**

Georgia

**Robert G. Hamilton**

United States of America

**Holger Heine**

Germany

**Martin Himly**

Austria

**Stephen Holgate**

United Kingdom

**Rose Kamenwa**

Kenya

**Amy Kantar**

Italy

**Monroe King**

United States of America

**Pierluigi Koch**

Italy

**Hans-Jørgen Malling**

Denmark

**Alberto Martelli**

Italy

**Marcus Maurer**

Germany

**Jim McKenna**

United Kingdom

**Martin Metz**

Germany

**Harold Nelson**

United States of America

**Robyn O'Hehir**

Australia

**Ayse Bilge Ozturk**

Turkey

**Pierluigi Paggiaro**

Italy

**Oscar Palomares**

Spain

**Hae-Sim Park**

South Korea

**Paola Parronchi**

Italy

**Giovanni Passalacqua**

Italy

**Carlos Pastor Vargas**

Spain

**Diego Peroni**

Italy

**Jay Portnoy**

United States of America

**Lars K. Poulsen**

Denmark

**David Price**

United Kingdom

**Kaisa Pyrhönen**

Finland

**Nelson Rosario**

Brazil

**Hugh Sampson**

United States of America

**Mario Sanchez-Borges**

Venezuela

**Joaquin Sastre**

Spain

**Glenis Scadding**

United Kingdom

**Irma Schabussova**

Austria

**Nicola Scichilone**

Italy

**Gianenrico Senna**

Italy

**James Sublett**

United States of America

**Stanley Szefler**

United States of America

**Mimi Tang**

Australia

**Alberto Tedeschi**

Italy

**Michael Wechsler**

United States of America

**Christina West**

Sweden

**Andrew White**

United States of America

**Torsten Zuberbier**

Germany

